# Clinical characteristics of rheumatoid arthritis patients undergoing cervical spine surgery: an analysis of National Database of Rheumatic Diseases in Japan

**DOI:** 10.1186/1471-2474-15-203

**Published:** 2014-06-13

**Authors:** Shurei Sugita, Hirotaka Chikuda, Yuho Kadono, Hiroshi Ohtsu, Katsushi Takeshita, Jinju Nishino, Shigeto Tohma, Sakae Tanaka

**Affiliations:** 1 The Department of Orthopaedic Surgery, The University of Tokyo, Hongo 7-3-1, Bunkyo, Tokyo, Japan; 2 The Center for Lifetime Cancer Education, Juntendo University, Hongo 2-1-1, Bunkyo, Tokyo, Japan; 3 Orthopaedic Surgeon, Nishino Clinic Orthopedics and Rheumatology, Nishigaoka 2-9-15, Kita, Tokyo, Japan; 4 The Department of Rheumatology, Clinical Research Center for Allergy and Rheumatology, Sagamihara National Hospital, National Hospital Organization (NHO), Sakuradai 18-1, Sagamihara, Kanagawa, Japan

**Keywords:** Rheumatoid arthritis, Cervical spine surgery, Database

## Abstract

**Background:**

The aim of this study was to examine the clinical characteristics of rheumatoid arthritis (RA) patients who underwent cervical spine surgery using a multicenter observational database.

**Methods:**

We obtained data from a nationwide observational cohort database of patients with rheumatic diseases (National Database of Rheumatic Diseases by iR-net in Japan (NinJa)) for the fiscal years 2003 to 2011. A total of 39 out of 60 patients who underwent cervical spine surgery for a RA-related cause and whose data were available for two consecutive years (to assess the preoperative patient status) were chosen as cases. Patients with a non-RA-related cause of surgery (e.g., trauma) were excluded. First, we compared the patient characteristics between the cases and total patients in the same fiscal year. Next, 106 eligible controls, who were defined as RA patients enrolled in the same fiscal year as the case subjects, who were matched for age, gender and disease duration (within ±1 year), were selected. We compared the demographic data between the two groups. We also calculated the percentage of patients who underwent cervical spine surgery (surgeries/total number of patients) in fiscal years 2003 to 2011.

**Results:**

Although the proportion of patients using biologics linearly increased during study period, the percentage of patients undergoing cervical spine surgeries remained unchanged, at approximately 0.15%. These cases had more tender joints (3 vs. 1, p < 0.01) and exhibited a significantly higher Modified Health Assessment Questionnaire (MHAQ) score (1.13 vs. 0.5, p < 0.01), C-reactive protein (CRP) (1.5 vs. 0.36, p < 0.01), and disease activity score (DAS) 28-CRP (3.63 vs. 2.81, p < 0.01) compared to the controls.

**Conclusions:**

Our study revealed that RA patients requiring cervical spine surgery have a higher disease activity (as represented by the DAS28-CRP) and are more functionally disabled (as represented by the MHAQ) than control patients.

## Background

Rheumatoid arthritis (RA) is a chronic inflammatory disease that affects the synovial joints. Immunologic dysfunction results in the hypertrophy of the synovial tissue, causing erosion of the articular cartilage and subchondral bone [1]. The involvement of the cervical spine is common in RA patients, with a reported prevalence of up to 86% [2]. The majority of RA patients with radiological abnormalities of the cervical spine remain asymptomatic for years [3]. Only a small fraction of patients eventually require surgical treatment, primarily due to neurological deterioration. For those who need surgical intervention, early surgical intervention is desirable [2,4]. If there is a delay in surgical intervention, it can lead to a poor outcome and even death [5,6]. Neurological improvement can be seen after surgery; in one study, patients with RA and patients with osteoarthritis had similarly high proportions of good global scores and satisfaction with their treatment after surgery [7]. However, it remains unclear whether RA patients undergoing cervical spine surgery differ from those who do not receive surgical treatment in terms of their clinical characteristics and the disease activity of RA. A previous cohort study showed a correlation between the severity of the RA activity in the peripheral joints and the progression of cervical spine instability [8]. However, few studies using a large observational database have examined this issue.

The aim of this study was to examine the clinical characteristics of RA patients who underwent cervical spine surgery using a multicenter observational database. Our hypothesis is that RA patients requiring cervical spine surgery have a higher disease activity than those who do not undergo surgical treatment.

## Methods

### Data source

The National Database of Rheumatic Diseases by iR-net in Japan (NinJa) is a nationwide, multicenter (40 centers), observational cohort database of patients with rheumatic diseases established in 2002 in Japan [9]. The collected data consist of two components: patient information over the course of the year [outcome, death, hospitalization, operations, number of total joint arthroplasty procedures in large joints (hip, knee, shoulder, elbow), malignancy and tuberculosis] and information collected on each day that the patient visits the outpatient department of each center [tender-joint count (TJC) and swollen-joint count (SJC), modified health assessment questionnaire (MHAQ) score, Steinbrocker functional classification (class), patient global and pain visual analog scale scores (PtGVAS, PtPainVAS), the doctor VAS (DrGVAS), erythrocyte sedimentation rate (ESR), C-reactive protein (CRP) level, disease activity score (DAS) 28-CRP and the use of corticosteroids, methotrexate (MTX) or biologics].

### Patients

The data of RA patients who were enrolled in the fiscal years from 2003 to 2011 were employed for the analysis (the fiscal year was defined as April to March, because official schedule tends to be run according to the fiscal year in Japan). The data acquired from 2002 was eliminated because of the incompleteness of data. All patients fulfilled the 1987 classification criteria of the American College of Rheumatology. After simple comparison of the demographic data year by year, the patients who underwent cervical spine surgery (excluding those who underwent cervical spine surgery for non-RA-related causes) and whose data were available for two consecutive years were chosen as cases. The total number of cases included was 39.

The background data of the cases were extracted from the fiscal year before surgery to represent the preoperative status. We first simply compared the background data of the patients between the cases and total patients enrolled in the same fiscal year. Then, eligible controls (1:3 pair matching) were selected, which were defined as patients enrolled in the same fiscal year as the case subjects, who were matched for age (within ±1 year), gender and disease duration (within ±1 year). The total number of controls was 106. Some cases did not have three controls. The background data of the cases in the previous year of surgery and the controls were compared. The examined factors were the stage, class, MHAQ, CRP, ESR, PtPainVAS, PtGVAS, DrGVAS, DAS28-CRP, number of arthroplasty procedures, tender-joint count and the use of steroids, MTX or biologics.

The NinJa project was reviewed and approved by the National Hospital Organization research ethics committees, and this study protocol was approved by the research ethics committees of the Sagamihara National Hospital and the University of Tokyo Hospital, and all enrolled patients provided their written informed consent.

### Analyses and statistics

The JMP 11.0.0 software program (SAS, Cary, NC) was used for the statistical analysis. Descriptive statistics were used to analyze the clinical information, demographic factors and other test data. Continuous variables were expressed as the medians and interquartile ranges (IQR). Yearly trends were analyzed by a single regression model or chi-square test, or by the Tukey-Kramer method. Differences between groups were examined using the Mann–Whitney U test for continuous variables or the chi-square test for categorical data, when appropriate. A p-value <0.05 was considered to indicate a significant difference.

## Results

Table 1 shows the demographic and disease characteristics of the patients included in the NinJa Database. There were no significant yearly differences in gender (chi-square test). However, the age was significantly older in 2010 (p < 0.01, Tukey-Kramer method) and the disease duration was significantly shorter in fiscal year 2007 (p < 0.01, Tukey-Kramer method) (Table 1). The proportion of patients using corticosteroids decreased from 2003 to 2011, whereas the proportion using MTX and biologics linearly increased during this period (coefficient: 2.43, p < 0.01 for MTX, coefficient 2.76, p < 0.01 for biologics, single regression model) (Table 1). We also counted the number of patients who underwent cervical spine surgery or total joint replacement and those with RA recruited in this database in each fiscal year. The annual incidence of cervical spine surgery was approximately 0.17%, and remained relatively stable during the study period, whereas that of total joint replacement showed a slightly decreasing trend (coefficient -0.21, p < 0.01 for total knee replacement, coefficient -0.05, p < 0.01 single regression model) (Table 1).

**Table 1:** Demographic and desease characteristics of the patients included in NinJa database

**Fiscal year**	**2003**	**2004**	**2005**	**2006**	**2007**	**2008**	**2009**	**2010**	**2011**	**p-value**	**Coefficient**
Total number of registered patients	4100	3994	4650	5152	5463	6502	7179	7254	10368		
Age (years) (IQR)	62(55–69)	63(55–70)	64(56–71)	63(56–71)	64(56–71)	64(56–71)	64(56–72)	65(57–72)	65(56–72)	<0.0001	
Gender (female:male)	3410:700	3313:681	3832:818	4229:923	4448:1015	5346:1156	5895:1284	5921:1333	8375:1993	0.7341	
Disease dutaion (years) (IQR)	11(5–20)	11(5–19)	10(5–19)	10(5–19)	9(4–19)	10(5–19)	10(5–19)	10(5–19)	9(4–18)	0.0020	
Use of MTX (%)	36	48	53	53	47	51	64	58	60	0.0084	2.43
Use of corticosteroid (%)	64	63	62	63	61	58	53	53	49	0.0002	-1.88
Use of biologics (%)	0.4	2.1	4.3	9	12	14	20	19	20	<0.0001	2.76
Cervical spine surgery (cases) (%)	8 (0.19)	7 (0.17)	4 (0.08)	6 (0.12)	8 (0.15)	10 (0.15)	10 (0.14)	15 (0.21)	30 (0.29)	0.1593	0.01
Total knee replacement (cases) (%)	146 (3.55)	121 (3.02)	117 (2.52)	130 (2.52)	132 (2.42)	118 (1.81)	156 (2.17)	133 (1.87)	158 (1.52)	0.0002	-0.21
Total hip replacement (cases) (%)	33 (0.80)	37 (0.93)	50 (1.08)	48 (0.93)	45 (0.82)	51 (0.78)	46 (0.64)	43 (0.59)	56 (0.54)	0.0098	-0.05

The data are represented as the medians (IQR). The yearly differences were analyzed by the Tukey-Kramer method.

Yearly trends were analyzed by a single regression model, and are expressed by coefficients and p-values.

Fusion surgery was the most frequently performed surgery (29 cases, 74.4%), followed by laminoplasty (four cases, 10.25%), laminectomy (two cases, 5.1%) and unspecified surgery (four cases, 10.25%).

Table 2 summarizes the patient characteristics, which compared the cases and total patients enrolled in the same fiscal year. The data showed that the cases tended to be more functionally disabled (e.g. MHAQ, PtGVAS) and to have a more severe RA status (e.g. number of arthroplastic surgeries, tender joint count, swollen joint count, CRP, ESR, DAS28-CRP) and to have a longer disease duration. However, RA is known to be progressive disease, and there was a possibility that a longer disease duration could have influenced the other parameters (function, RA severity). Therefore, we decided to compare the characteristics of the patients using a case-matched study, as described in the Methods.

**Table 2:** Summary of patient characteristics

**Fiscal year**	**2004**		**2005**		**2006**		**2007**		**2008**		**2009**		**2010**	
	**Cases**	**Total**	**Cases**	**Total**	**Cases**	**Total**	**Cases**	**Total**	**Cases**	**Total**	**Cases**	**Total**	**Cases**	**Total**
Age (years) (IQR)	58.5(55.5-72)	63(55–70)	65(62–76)	63(55–70)	60(59–76)	63(56–71)	61(59–63)	64(56–71)	64	64(56–71)	63(58–74)	64(56–72)	65(57–70)	65(57–72)
Gender (female:male)	3:1	3313:681	2:1	3832:818	3:0	4229:923	2:0	4448:1015	1:0	5346:1156	11:2	5895:1284	13:0	5921:1333
Disease dutaion (years) (IQR)	6(4–9)	11(5–19)	15(12–36)	10(5–19)	13(11–30)	10( 5–19)	23.5(15–32)	9(4–19)	24	10(5–19)	16(12–20)	10(5–19)	20(15–40)	10(5–19)
Arthroplastic surgery (IQR)	0(0–0)	0(0–0)	1(0–2)	0(0–0)	1(0–4)	0(0–0)	2(0–3)	0(0–0)	0%	0(0–0)	0(0–3)	0(0–0)	0(0–1)	0(0–0)
Tender joint count (IQR)	9(1–30)	3(1–6)	15(13–16)	3(1–6)	1(0–4)	2(1–6)	19(4–34)	2(0–5)	4	2(0–5)	3(1–9)	2(0–4)	2(1–4)	1(0–3)
Swollen joint count (IQR)	2(0–10)	2(0–5)	0(0–4)	2(0–5)	0(0–2)	1(0–4)	3(3–3)	1(0–3)	3	1(0–4)	2(0–6)	1(0–3)	2(0–5)	1(0–3)
MHAQ (IQR)	1.4(0.35-1.48)	0.5(0–1)	2.3(1.3-2.9)	0.4(0–1)	1.5(0.88-2)	0.4(0–1)	1.55(1–2.1)	0.4(0–1)	1.25	0.38(0–1)	1(0.69-1.44)	0.25(0–0.9)	1(0.26-1.94)	0.25(0–0.9)
CRP (mg/dl) (IQR)	3.4(0.9-4.2)	0.59(0.18-1.7)	2.7(1.75-3.35)	0.55(0.18-1.57)	0.47(0.05-0.71)	0.47(0.18-1.4)	1.26(1.02-1.5)	0.41(0.17-1.22)	1.95	0.31(0.13-1.03)	1.04(0.18-2.78)	0.28(0.11-0.9)	2.0(0.14-3.5)	0.26(0.1-0.76)
ESR (mm/h) (IQR)	38.5(14–66)	37(20–60)	43(17–77)	35(19–56)	23(9–46)	33(18–55)	74.5(46–103)	31(16–51)	51	29(15–49)	37.5(29–71.3)	28(14–47)	55.5(5.5-84.8)	25(13–45)
DAS28-CRP (IQR)	4.15(3.15-6.11)	3.34(2.45-4.25)	4.71(4.67-5.38)	3.35(2.43-4.24)	2.98(1.13-3.61)	3.14(2.29-4.06)	4.93(3.46-6.39)	2.99(2.12-3.89)	4.19	2.85(2.04-3.75)	3.32(2.58-4.46)	2.72(1.94-3.6)	3.39(2.3-4.78)	2.55(1.83-3.46)
Stage1 (%)	0	14.5	0	15	0	15.9	0	17.7	0	16.6	8.3	18	7.7	19.6
Stage2 (%)	100	25	33.3	25.2	0	25	0	25.7	0	25.6	0	27.2	0	26.5
Stage3 (%)	0	21	33.3	20.3	0	19.9	50	20.4	0	20.7	25	19.3	15.4	19.6
Stage4 (%)	0	39.5	33.3	39.5	100	39.2	50	36.2	100	37.1	66.7	35.5	76.9	34.3
Class1 (%)	0	25	0	26.2	0	26.2	0	26.9	0	25.3	25	26	0	28.2
Class2 (%)	75	52.7	0	53.4	33.3	53.3	0	52.9	0	52	50	53.9	61.5	52.6
Class3 (%)	25	19.1	66.7	17.4	66.7	17.4	100	17.5	0	19.3	25	16.8	38.5	16.4
Class4 (%)	0	3.2	33.3	3	0	3.1	0	2.7	100	3.4	0	3.3	0	2.8
PtPainVAS (IQR)	5.5(2.3-7.0)	3.3(1.6-5.5)	6.1(4–7)	3.1(1.5-5.3)	5.5(1.6-6.8)	3(1.4-5.2)	5.5(2.5-8.5)	3(1.3-5.1)	4.3	2.6(1.1-5)	3.4(1.4-8.3)	2.6(1.1-5)	2.25(0.98-4.95)	2.4(1–4.8)
PtGVAS (IQR)	5.7(3–8.4)	3.5(1.7-5.5)	6.8(2.3-8.1)	3.4(1.6-5.3)	5.9(0.2-7.8)	3.2(1.5-5.2)	5.5(2.5-8.5)	3(1.4-5.1)	5.3	2.8(1.2-5)	4.7(0.85-7.75)	2.7(1.2-5)	3.1(1.85-5.9)	1.6(0.7-2.9)
DrGVAS (IQR)	7(3.4-8.3)	2.5(1.3-4.1)	5.2(3.1-7)	2.4(1.1-4)	0.7(0.5-3.4)	2(1–3.5)	6.15(3.3-9)	1.8(0.9-3.1)	2.6	1.6(0.6-3)	3.8(0.8-4.8)	1.8(0.8-3)	2.5(1.05-4.4)	0.25(0–0.88)
Use of MTX (%)	33	48	0	53	100	53.9	50	47.3	0	51.1	53.8	64.3	0	58.2
Use of corticosteroid (%)	100	63	100	62	100	64	100	61	100	59.1	100	53	84.6	53.4
Use of biologics (%)	0	2.1	0	4.3	0	9	0	11.7	0	13.6	38.5	20	0	18.6

VAS: visual analog scale, MHAQ: Modified Health Assessment Questionnaire, CRP: C-reactive protein, ESR: erythrocyte sedimentation rate, DAS28: Disease Activity Score of 28 Joints, MTX: Methotrexate.

A comparison of the cases and total number of patients enrolled in the same fiscal year. The data are presented as the medians (IQR).

Table 3 summarizes the patient characteristics according to whether they underwent cervical spine surgery. The patients who underwent cervical spine surgery were more likely to have more tender joints (3 vs. 1, p < 0.01). They also exhibited significantly higher MHAQ scores (1.13 vs. 0.5, p < 0.01), higher DAS28-CRP values (3.63 vs. 2.81, p < 0.01), a higher PtGVAS (4.9 vs. 2.9, p < 0.05) and a higher DrGVAS (3.4 vs. 1.6, p < 0.05). They also tended to be in higher Steinbrocker functional classification (p < 0.05). Regarding the treatment of RA, the use of MTX was significantly lower in cases (13% vs. 40%, p < 0.01), and use of corticosteroids was significantly higher in cases (90% vs. 60%, p < 0.01) than controls. The use of biologics was not significantly different between cases and controls.

**Table 3:** Comparison of the background data of the cases and controls

	**Cases**	**Controls**	**p value**
Age (years) (IQR)	63(59–73)	63(59–69)	0.7224
Gender (female:male)	35:4	99:7	0.4614
Disease dutaion (years) (IQR)	15(12–30)	15(11–22)	0.52
Arthroplastic surgery (%)	41	31	0.1527
Tender joint count (IQR)	3(1–10)	1(0–4)	0.0087
Swollen joint count (IQR)	2(0–4)	1(0–3)	0.1389
MHAQ (IQR)	1.13(0.75-1.88)	0.5(0.1-1)	0.0001
CRP (mg/dl) (IQR)	1.5(0.26-3.05)	0.36(0.13-1.06)	0.0023
ESR (mm/h) (IQR)	46(21–70)	33(20.5-57.5)	0.1948
DAS28-CRP (IQR)	3.63(2.60-4.68)	2.81(2.01-3.43)	0.0012
Stage1 (%)	5.3	6.5	
Stage2 (%)	13.2	19.6	
Stage3 (%)	18.4	19.6	
Stage4 (%)	63.1	54.3	0.7823
Class1 (%)	7.9	20.4	
Class2 (%)	47.3	58.1	
Class3 (%)	39.5	18.3	
Class4 (%)	5.3	3.2	0.0395
PtPainVAS (IQR)	3.9(1.8-6.4)	3(1.2-5)	0.0937
PtGVAS (IQR)	4.9(2–6.8)	2.9(1.2-5)	0.0314
DrGVAS (IQR)	3.4(1.4-5.1)	1.6(1–3.1)	0.0121
Use of MTX (%)	13	40	0.0022
Use of corticosteroid (%)	90	60	0.0007
Use of biologics (%)	10	8	0.7414

The data are presented as the median (IQR). P values were calculated using the chi-square test for gender, Steinbrocker functional class and stage and arthroplastic surgery. The Mann–Whitney U test was used for the other items.

## Discussion

In this retrospective analysis using a large database, we found that only a small fraction (1.7/1,000/year) of patients underwent cervical spine surgery. However, despite the increasing use of biologics, the incidence of cervical spine surgery remained unchanged over recent years. Our data also showed that patients undergoing cervical spine surgery had a higher disease activity and exhibited a more severe degree of functional disability than those who did not undergo cervical spine surgery.

Although radiographic abnormalities of the cervical spine are frequently seen in RA patients [2], our study showed that only a small fraction (1.7/1,000/year) of patients had a history of cervical spine surgery. A previous study showed that the number of RA-related cervical spinal surgeries in 609 RA patients was 0.2%, which was similar to our data [10]. There was a stark discrepancy between the high prevalence of radiographic abnormalities and the relative rarity of cervical spine surgery. There are several possible explanations for this discrepancy. One is that the prognosis of RA patients with cervical lesions is generally favorable. For example, a previous report showed that there was cervical spine involvement in up to 86% of RA patients, but only 10% of these patients actually developed neurological deterioration [11]. However, it is also possible that some patients were underdiagnosed, presumably due to the involvement of multiple joints.

The annual incidence of cervical spine surgery did not change much during recent years according to the NinJa database. The increased use of biologics has reportedly led to a reduction in the number of RA-related arthroplasty procedures, such as total hip replacements [12]. Our data agreed with this previous study, showing a decrease in the number of total joint replacement surgeries; however, the number of cervical spine surgeries did not exhibit a substantial decrease. We hypothesize that the protective effects of biologics on the cervical spine are limited. In line with this view, a prior study showed that the use of biologics prevented the development of de novo cervical spine lesions in RA patients, but failed to inhibit the progression of preexisting RA lesions [13]. Regarding the use of MTX, our data showed that the use of MTX was significantly lower in cases than controls. Another previous study showed that the use of MTX did not improve the cervical spine instability [14], so our results are reasonable. On the contrary, the use of corticosteroids was significantly higher in cases than in controls. A previous study showed that the administration of a higher dose of corticosteroid was a significant general factor predicting the development of myelopathy [15], supporting our present findings.

We also found that patients undergoing cervical spine surgery had a high disease activity despite receiving intensive treatment for RA, including biologics. There was a previous study that showed a correlation between the progression of peripheral erosive disease and the severity of cervical subluxation [16]. Our data further supported that study by using a case-matched method. Some previous reports recommended early prophylactic surgical intervention for cervical instability in RA patients [17-19]. Regardless whether surgery is undertaken, patients with a high disease activity should be closely monitored for neurological deterioration.

Interestingly, there were no significant differences in the pain scores between those with and without a history of cervical spine surgery. Therefore, physicians should pay close attention to not only the patient’s pain, but also the neurological status and function of patients who do not report high pain levels.

There are several possible limitations associated with this study. First, we were unable to conduct a multivariate analysis because the number of patients who underwent cervical spine surgery was too small. In addition, we were unable to obtain detailed information on the type of spinal lesions (e.g. atlantoaxial subluxation) and the surgical procedures. We also could not count the number of cases with cervical involvement because we did not take plain radiographs of the cervical spine in every patient. Therefore, we could not determine how many of the RA patients in the NinJa database had cervical involvement. Finally, the institutions participating in the NinJa database are mostly national hospitals, which may limit the generalizability of our findings. In addition, our research period was relatively short, so our results regarding the effects of biologics on the cervical spine may change in the future as more patients are treated using these agents.

## Conclusions

Our findings revealed that RA patients requiring cervical spine surgery tend to have a higher disease activity than control subjects. Our results also indicate that conducting a meticulous evaluation of cervical lesions is therefore required, particularly in patients with a high disease activity in spite of the intensive use of biologics.

## Competing interests

Each author certifies that they have no commercial associations that might pose a conflict of interest in connection with the submitted article.

## Authors’ contributions

SS, HC, JN and ST contributed to the conception and design of the study. SS, YK, HO, KT and ST contributed to the analysis, and all authors contributed to the interpretation of the results. SS drafted the article; all authors revised it critically and approved the final version submitted for publication. All authors read and approved the final manuscript.

## Pre-publication history

The pre-publication history for this paper can be accessed here:

http://www.biomedcentral.com/1471-2474/15/203/prepub
